# Experimental Study on the Transport and Alteration Behavior of Aerosols From Low Density Powders for Acute Inhalation Toxicology Studies

**DOI:** 10.3389/fpubh.2022.907202

**Published:** 2022-06-17

**Authors:** Benno Wessely, Michael Stintz, Juergen Nolde, Otto Creutzenberg

**Affiliations:** ^1^Institut für Verfahrenstechnik und Umwelttechnik, Technische Universität Dresden, Dresden, Germany; ^2^Grace Europe Holding GmbH, Worms, Germany; ^3^Department of Inhalation Toxicology, Fraunhofer Institute for Toxicology and Experimental Medicine (Fh-ITEM), Hannover, Germany

**Keywords:** acute inhalation toxicology, pyrogenic powder aerosols, aerosol transport, particle size, MMAD, aerosol measurement, laser diffraction

## Abstract

Low density powders have a bulk density of less than 100 kg/m3 and are produced technically by flame pyrolysis of silicon tetrachloride (pyrogenic powders such as pyrogenic silica) or wet-chemically by sol-gel processes (e.g. silica-gel) or precipitation reactions using sodium silicate solution and a mineral acid. The transport and alteration behavior of aerosols from low density powders was investigated in a device for toxicological inhalation studies. The test conditions corresponded to those for acute toxicology studies according to OECD Guideline 436. The use of cascade impactors, required by guideline, has not proven successful for low density powders as the fragile agglomerate structures are destroyed during the measurement. As an alternative and non-invasive measurement method, laser diffraction spectroscopy has proved very successful in the present investigations. In particular, aerosols from pyrogenic powders of low density showed a distinctive tendency to re-agglomerate, especially at concentrations >500 mg/m^3^mm^3^. Investigation results indicate that aerosol particle size must be monitored over the entire acute inhalation test period for acute inhalation studies to be performed reliably.

## Introduction

For particulate systems with low bulk or tapped density, it is difficult to achieve a specific stable target aerosol concentration to be used in acute inhalation toxicity testing without altering and deposition. Thus, the OECD Guideline 39 states that “”.... *elaborate pre-tests without animals may be needed to achieve a specific temporarily stable atmosphere concentration and particle size distribution* [(1), p. e2–e4]. The particle behavior without such a pretest is unpredictable, in particular the tendency for aerosol altering, i.e., agglomeration and precipitation in the test chamber, which can result in misleading or inconsistent results in the animal studies according to OECD Guidelines 403 and 436. Previous animal studies mention difficulties with the atmosphere generation and different maximal technically achievable concentrations for particular low-density systems, in some cases, even with the same substance/grade when different atmosphere generation technologies were applied. Furthermore, OECD Guideline 39 states in paragraph 51: *It can be difficult or*

*impossible to generate a respirable (MMAD of 1-4* μ*m) liquid or solid aerosol at this concentration without encountering experimental shortcomings. As aerosol concentration increases, particle size also increases due to the aggregation of solid particles or coalescing of liquid particles. The usual consequences are (1) a decrease in the respirable particle size fraction (and thus reduced toxicity), (2) increased fluctuation and variability in inhalation chamber concentrations accompanied by increased spatial inhomogeneities, (3) overloading of equipment used to characterize test atmospheres, and (4) a divergence of nominal and actual concentrations. At very high concentrations, dry powder aerosols and chemically reactive liquid aerosols (e.g., polymers) tend to form conglomerates in the proximal nose causing physical obstruction of the animals' airways (e.g., dust loading) and impaired respiration which may be misdiagnosed as a toxic effect* [(1), p. 37].

To provide a standardization of this pretest suggested by OECD Guideline 39, a characterization method was investigated that included aerosol generation, particle size measurement at the point of generation, point of delivery, mass flow correlation and influence of standard particle size measurement equipment in relation to the specific physical chemical parameters of several test materials with low toxicity and low density. Aerosol behavior can have, as already mentioned in the respective guideline, a significant impact on the results of acute inhalation tests and lead to severe misinterpretation.

For acute inhalation studies according to the OECD Guideline 436, aerosols of powdered substances must be generated and available at the point of delivery over a period of 4 h, with a mass medium aerodynamic diameter (MMAD) of 1–4 μm and a geometric standard deviation σ_g_ of 1.5–3 (2).

According to the OECD concentration specifications and CLP regulations, (3) the required test concentrations are 5,000, 1,000, 500 mg/m^3^, or up to 50 mg/m^3^m in the tiered test program for the classification and categorization of particulates. In addition to mass concentration, the volumetric flow rate and particle size distribution (PSD) of aerosols at the point of delivery are specified. The mass particle size distribution is thereby provided as the distribution of the aerodynamic diameter x_ae_, i.e., the particle density is defined at 1,000 kg/m3. The median value of this PSD is the above mentioned MMAD. During the test, the particle concentration and particle size distribution are measured and documented at prescribed frequencies. Recommended measurement methods are gravimetric concentration determination using an absolute filter and for the particle size distribution (PSD), aerodynamic measurement methods such as cascade impactors or aerodynamic particle sizers.

In the past, conflicting test results have indicated that greater attention must be paid to the particle size distribution and stability of the aerosol atmosphere at the point of delivery. Even if the aerosol generator runs within the required aerosol size specification, changes in PSD and concentration often occur during the aerosol flow through piping and distribution systems to the point of delivery. This is caused by effects such as agglomeration, wall buildup and associated segregation/precipitation phenomena. These effects are strongly influenced by particle concentration, particle properties (size, effective density, shape, surface properties, surface charge), and the conditions in the test apparatus.

Particularly critical products in this respect are aerosols from so-called low-density powders, which have a bulk density of less than 100 kg/m3. In this publication, the PSDs of a characteristic selection of powders were investigated at the inlet and after passing through a complete inhalation apparatus as a function of the operating time. The investigations were carried out at two concentrations in each case, a “maximum” particle concentration in the range of 4,500–7,000 mg/m3 and a “technically feasible” concentration for most powders of about 500 mg/m^3^.

Due to the special particle properties of the aerosols, the PSD could not be measured with a cascade impactor. The agglomerates formed in the airstream are very sensitive to shear forces and thus the shear forces induced by the cascade impactor change the particle size. Laser diffraction spectroscopy was selected and used as an alternative measurement method. Consequently, it was necessary to convert the aerosol specification based on the aerodynamic diameter into a geometric diameter distribution of the aerosols for each powder investigated. Unlike the aerodynamic diameter, the geometric diameter can be directly related to the geometric dimensions of the respiratory tracts of the test animals. The low dynamic and thus shear forces applied to any particles in the rat nose is based on the low flow velocity in the nasal cavities which was examined and modeled by Shang et al. 2015 (4). In addition, the geometric diameter of the low-density powders studied in this publication is up to four times larger than the aerodynamic diameter.

The results of the animal test are subject of a seconds publication, Krüger et al. Physical Obstruction of Nasal Cavities with Subsequent Asphyxia, causes lethality of Rats in an Acute Inhalation Study with Hydrophobic HMDZ Surface- Treated Synthetic Amorphous Silica (SAS) (under preparation).

In conclusion, essential corrective actions are recommended to ensure guideline-compliant aerosol atmosphere generation for acute inhalation studies.

## Fundamentals

### Aerosol Characteristics and Specifications According to OECD Guideline 436

#### Aerosol Characteristics

The relevant properties of aerosols can be described with the number or mass concentration of the particles and with the volume-related or number-related particle size distribution in general. Technical applications of aerosols generally involve the generation of the desired aerosol from powder, transport to the point of application and the application itself. At the point of delivery, the aerosol should be present for a constant time at the desired concentration and particle size distribution.

Both the aerosol concentration and particle size distribution (PSD) of low density powders undergo dynamic changes after the generation.

For laminar flow conditions, Marshall investigated these effects with a two-dimensional soft-sphere discrete-element simulation as a function of particle size and particle interaction forces. Among other things, it could be shown that for the same particle concentration, the effects of wall bonding and agglomeration strongly depend on the particle-particle interaction forces (5).

In addition, phenomena such as classifying effects, sedimentation, wall bonding or the debonding and redispersion of deposits back into the aerosol atmosphere must also be considered.

The challenge of the temporal and local change of powder aerosols is therefore relevant for all applications and, as necessary, must be investigated metrologically.

#### Acute Inhalation Toxicity Test Specifications According to OECD Guideline 436

**Figure 1:**
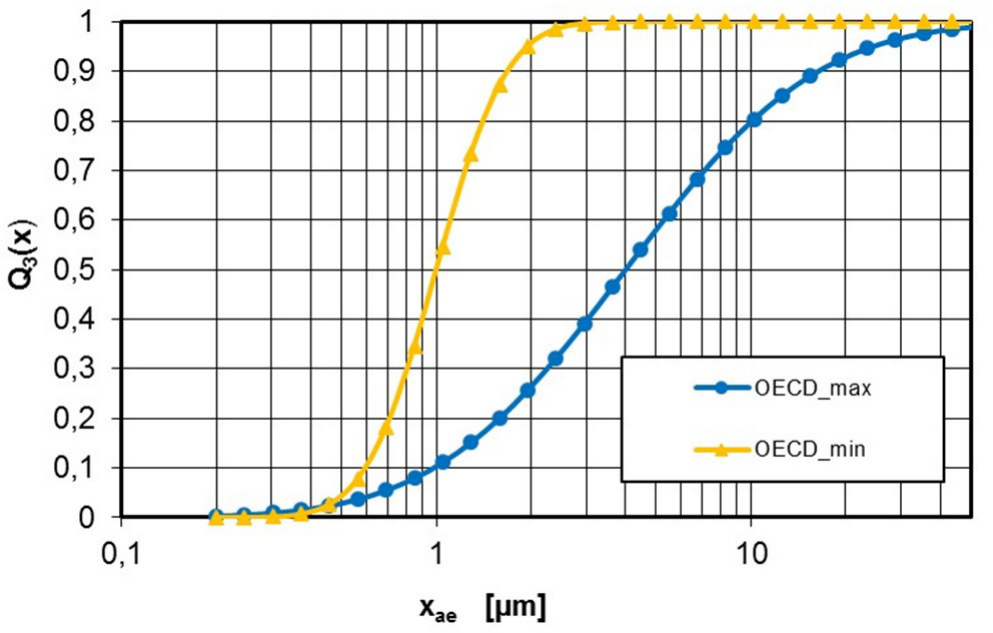
Range of aerodynamic PSD according to OECD Guideline 436: OECD_min_ x_ae, 50_ = MMAD = 1 μm, σ_g_ = 1.5; OECD_max_ x_ae, 50_ = MMAD = 4 μm, σ_g_ = 3.

In this publication, the transport behavior of powder aerosols is investigated as it applies to inhalation toxicology studies according to OECD Guideline 436 (2). Acute inhalation studies are part of the mandatory data set for powdery substances according to REACH Annex VIII (6). In acute inhalation studies according to OECD Guideline 436, rats are exposed for 4 h to an aerosol concentration in a range of 50–5,000 mg/m^3^ (graded at 50, 500, 1,000, and 5,000 mg/m^3^). The particle size distribution of the aerosol particles at the point of delivery (exposure port of the inhalation chamber) is specified with an aerodynamic diameter of x_ae, 50_ = MMAD = 1–4 μm with a permissible geometric standard deviation of σ_g_ = 1.5–3. The MMAD describes the 50% quantile of the mass-wise cumulative distribution Q_3_(x) of the aerodynamic diameter x_ae_. In the case of a logarithmic normal distribution, this results in limit curves for the aerodynamic diameter according to OECD Guideline 436 (Figure 1) (2).

Results from animal studies with this kind of inhalation testing devices and set-up showed unexpected results are sometimes obtained especially for low density powders. These include negative results with known toxic powders as well as positive results with expectedly safe powders. Although the aerodynamic PSD is determined during the cascade impactor tests, a correlation of these contradictory results with the particle size distribution at the point of delivery, the exposure port of the inhalation chamber, is suggested. Agglomerates could block the upper airways and, in certain cases, lead to suffocation of the test animal.

#### Need for and Qualification of Alternative Measurement Techniques for PSD Determination

Dry sand has a bulk density of about 1,200 kg/m3. In contrast, low density powders, such as pyrogenic, silica gel or precipitated amorphous SiO_2_O, often have a bulk density of less than 100 kg/m^3^. These powder aerosols have the following special features:

- low effective particle density and agglomeration tendency

- low density due to high porosity
- geometric diameter > aerodynamic diameter

- high agglomeration tendency

- large specific surfaces
- rough, form-fitting particle surfaces

- shear-sensitive, fragile particle structures
- In the case of surface treatment, the surfaces are not wettable and the large bulk volume is maintained even in the presence of liquids.

Guideline-compliant low-density powder aerosol agglomerates in the airflow in the micrometer range (single- to double-digit figures); they are very fragile and cannot be detected non-destructively with the prescribed aerodynamic methods (e.g., cascade impactor). The measurement procedures recommended in OECD Guideline 436 for determining aerodynamic PSD, such as cascade impactors or aerodynamic particle sizers (APS), can destroy fragile agglomerates due to internal mechanical stress (acceleration forces, shear forces in nozzles and hose lines) and thus significantly alter the aerosol state in the measurement result. Consequently, the cascade impactor measures a PSD shifted to finer particles at the point of delivery, which in fact does not exist.

Furthermore, at the required concentrations, APSs require aerosol dilution by a factor of 1,000, which is associated with additional mechanical stress on the particles in the injector system due to the addition air needed for the dilution. The measuring methods suggested cannot detect the larger agglomerates due to the upper limit of the measurement range. Corresponding test results for the use of cascade impactors are presented in the test results section.

Due to the concentration range and the expected maximum agglomerate sizes >30–50 μm at the point of delivery, a commercial laser diffraction spectrometer of the type Helos-KR (Sympatec GmbH) was used as an alternative measurement system. Particle concentrations from about 300 to 5,000 mg/m^3^ can be analyzed with the used measuring device without dilution. The open optical system of the instrument allows the PSD to be measured in free flow without contact and almost without mechanical stress. Dilution is unnecessary. The actual state of the aerosol almost without distortion can only be recorded in this way.

The particle sizes determined with a laser diffraction spectrometer represent physically the diameter of laser diffraction equivalent spheres. The particle size can be interpreted as geometric diameter, *x*. To allow comparison with the specification of OECD Guideline 436, the aerodynamic PSD “OECD_max_” in Figure 1 was converted to corresponding geometric PSD using Equation 1 for all powders.

Previous studies have shown that it is possible to convert aerodynamic diameters to laser diffraction measurement data using effective agglomerate densities (7). The relationship between the geometric diameter *x* and the aerodynamic diameter *x*_*ae*_ is described by Equation 1:


(1) $$\begin{array}{r} x=\sqrt{\frac{\rho_{1000}}{\rho_{eff}}}x_{ae}\;with\;\rho_{eff}=\rho_{s}\left(1-\varepsilon_{p}\right)\left(1-\varepsilon_{Agg}\right) \end{array}$$

ρ_1000_ = 1,000 kg/m^3^ and represents the reference density of the aerodynamic diameter (water). The effective particle density ρ_eff_ depends on the porosity of the particles ε_P_ and, in the case of agglomerates, additionally on ε_Agg_. For non-porous particles, ρ_eff_ takes the value of the solid's (skeleton) density ρ_s_. For pyrogenic powder systems (e.g., pyrogenic amorphous silica, alumina), the tamped density of the powder in case of strong dispersion can be used for ρ_eff_ (3). For the other powdered substances, values based on the effective particle density were applied. Using that assumption, it is possible to convert the OECD MMAD specification for each powder to the measured geometric diameters. The measurement results are presented as geometric diameter x, which can be firmly correlated with the MMAD of max. 4 μm required in OECD GD 39 and OECD TG 436 using the method explained above.

### Experimental Set-Up and Test Materials

#### Test Set-Up and Procedure

Based on experimental investigations on different synthetic amorphous silica and calcium carbonate powder aerosols, the changes in the aerosol atmosphere during aerosol transport through a nose-only-exposure system (inhalation chamber) for rodents are presented.

**Figure 2:**
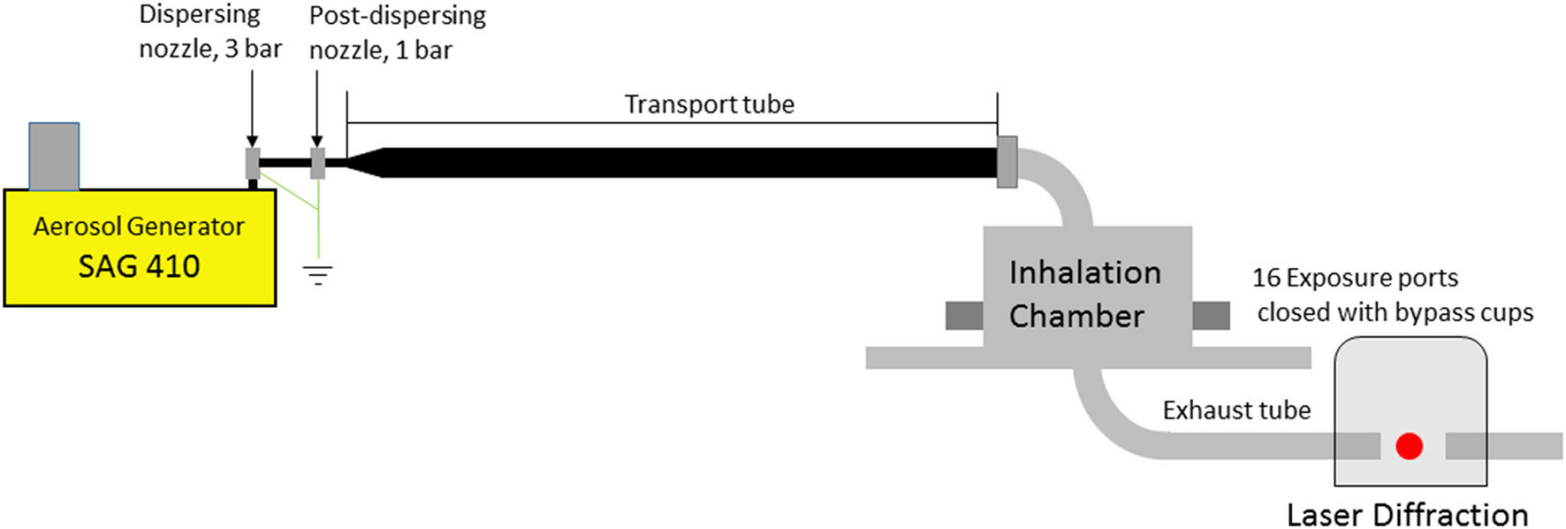
Test set-up for simulating inhalation experiments: aerosol generator and inhalation system, consisting of transport tube and inhalation chamber; laser diffraction particle sensor.

The chosen experimental set-up is based on equipment used at Fraunhofer ITEM Hannover for inhalation studies according to OECD 436 and other OECD Guidelines (8, 9). The main components are the inhalation chamber, the aerosol generator and a transport tube to the inhalation chamber. The experimental set-up is shown in Figure 2. Table 1 quantifies all essential details of the experimental set-up.

**Table 1:** Parameters of the test apparatus.

**Parameter**	**Value**
**Aerosol generator SAG 410**	
Dispersing pressure and feed rates Post-dispersing-nozzle pressure Air flow rate main nozzle and diluting nozzle nozzle driving air without secondary air from the environment Relative humidity measured at exposure unit	3 bar, feed rate 0 - 100% 1 bar 42 L/min ~20°C, 7 to −8% rel. humidity 25 to −40% rel. humidity
**Transport tube**	Length 1,500 mm, diameter 40 mm
**Inhalation chamber**	
Height/diameter of chamber segment Height / diameter of inner cylinder Chamber inlet diameter Exposure port nozzle diameter Exposure port outlet diameter Main exhaust diameter Number of exposure ports Air flow rate per port Exhaust flow rate	110 mm, 160 mm 60 mm, 125 mm 33 mm 6 mm 26 mm 22 mm 16 (closed with 16 bypass cups) 1 L/min 42 L/min
**Aerosol measurement**	
PSD with laser diffraction (Helos)	
-Exhaust - Ports - Duration for a measurement - Measuring range Purpose	No sampling, complete exhaust flow 4 ports, 4 L/min 10 mm tubes to instrument 30 s, 1 min (low opt. conc.) 0.9 to −175 μm PSD monitoring versus exposure time
Gravimetric mass concentration	
-Filter diameter - Sampling flow rate exhaust - Sampling flow rate at 4 ports - Min. sampled mass - Purpose	47 mm 40 L/ min 4 L/min 5 mg Mass conc. in the generator and behind the inhalation chamber

The particle size distribution of the aerosols was determined directly at the aerosol generator (input), at the exhaust tube and, in some cases, directly at four interconnected exposure ports of the inhalation chamber. Measuring the PSD at the exposure ports required sampling at four ports and sample transport through four 40-cm-long tubes with a diameter of 10 mm to achieve the minimum sample quantity for a measurement. This necessary procedure leads to additional changes in the aerosol condition. For this reason, the measurements were preferably performed directly at the outlet of the inhalation chamber. The aerosol condition at the ports and at the outlet are almost identical. To be able to estimate the particle losses in the system, the concentration at the above-mentioned locations was determined gravimetrically using a filter system.

Preliminary investigations have shown that the PSD of the aerosol can change significantly after passing through the test apparatus with increasing operating time. One reason for this is increasing particle deposits in the apparatus, which affect the agglomeration equilibrium of the aerosol (5). For this reason, the measurements were performed for different operating times, e.g., at the beginning, after 5, 10, 20 min, etc. The dispersion conditions at the aerosol generator and the volume flows through the system were constant (Table 1). The materials and the aerosol concentration varied. As mentioned above, two aerosol concentrations were investigated in each case, the high concentrations range from 4,500 to 7,300 mg/m3, within the range of maximum test concentrations. A range around 500 mg/m3 was selected for the seconds concentration. Preliminary investigations had shown that concentrations in this latter range represent those that are technically feasible without significantly altering the aerosol for most powders.

To investigate the effect of mechanical stress in a cascade impactor on PSD, 3 different powder aerosols were measured simultaneously using the laser diffraction spectrometer and cascade impactors. The impactor measurements were performed simultaneously with an ITEM impactor of the type Marple NS 298 at the point of delivery and with an impactor of the TU Dresden at the exhaust tube of the inhalation apparatus. This impactor was built for the particle size range 0.35–15 μm at the Martin Luther University Halle-Wittenberg, Department of Chemical Engineering, in Merseburg in 1985. The laser diffraction measurements were performed immediately before the impactor measurements.

To compare the measured particle size distributions at the outlet of the inhalation system with the OECD Guideline 436 requirements, the limiting curve “OECD_max_” (see Figure 1) was converted into a geometric particle size distribution *x* based on Equation 1. For pyrogenic powders, as mentioned above, the tapped density was used as the effective density, because the porosity of the flakes is nearly identical to that of the dispersed particles (7). For silica gel and calcium carbonate powders, this approach is not possible because the effective densities of the dispersed aggregates will be significantly different from those of the resulting agglomerates. Further investigation is needed to clarify these relationships. Nevertheless, in order to give a conservative estimate, the OECD_max_ curves are calculated based on the effective density of the aggregates. In the case of a very strong agglomeration, these curves will be too fine, i.e., more stringent than the OECD Guideline 436 MMAD specification.

#### Substance and Particle Characterization

The investigations were carried out on four powdered materials as shown in Table 2. The four investigated materials can be divided into two groups, pyrogenic amorphous silica powder and wet-chemically precipitated calcium carbonate and amorphous silica gel produced by a sol-gel process. The final particle size of the precipitated calcium carbonate and the amorphous silica gel is typically archived by milling or intensive milling, depending on the targeted PSD for the products.

**Table 2:** Products investigated, PSD at the aerosol generator, bulk and tamped density of powders.

**Substance**	**SiO_**2**_**	**SiO_**2**_**	**SiO_**2**_**	**CaCO_**3**_**
Substance type	Pyrogenic	Silica gel	Pyrogenic	Precipitated
Surface treatment	HDMS-treated	Untreated	Untreated	Untreated
Product	AEROSIL® R 812	SYLOID® 244 FP	HDK® N 20	SOCAL® UP-G
x_1_x_0;3_ [μm]	5.15	1.10	5.15	0.89
x_1_x_6;3_ [μm]	6.25	1.31	6.29	1.20
x_5_x_0;3_ [μm]	11.6	2.31	13.6	3.31
x_8_x_4;3_ [μm]	20.0	3.62	31.6	7.15
x_9_x_0;3_ [μm]	23.3	4.05	40.1	8.41
Bulk density [kg/m3]	40.8	61.0	32.5	226
Tamped density [kg/m3]	64.0	83.7	46.7	447
Eff. particle density [kg/m3]	64.0	242	46.7	1,000
x/x_ae_	4	2	4.6	1

In terms of particle structure, the powdered materials differ as shown below:

1. wet chemical powders (silica gel and calcium carbonate): stable, compact aggregates with a tendency to lower agglomeration;
2. pyrogenic powders (untreated and treated pyrogenic silica): fractal, aggregate-like particles of low effective density and high tendency to form large agglomerates; the agglomerates have very low shear stability (fragile).

The particle sizes given in Table 2 represent the state of the aerosol at the outlet of the SAG 410/U aerosol generator in which the powder particles are accelerated to about 50 m/s in an injection nozzle. These PSDs represent the aerosol state at the inlet of the transport tube of the inhalation apparatus. For pyrogenic powders such as fumed silica, the PSD represents an equilibrium state depending on the dispersion energy (10).

## Results

### PSD—Cascade Impactor vs. Laser Diffraction

In the first section, cascade impactor measurements and laser diffraction analysis results using three powders as examples will be compared and discussed. For this purpose, it was necessary to convert results of the impactor measurements into geometric diameters using Equation 1.

**Figure 3:**
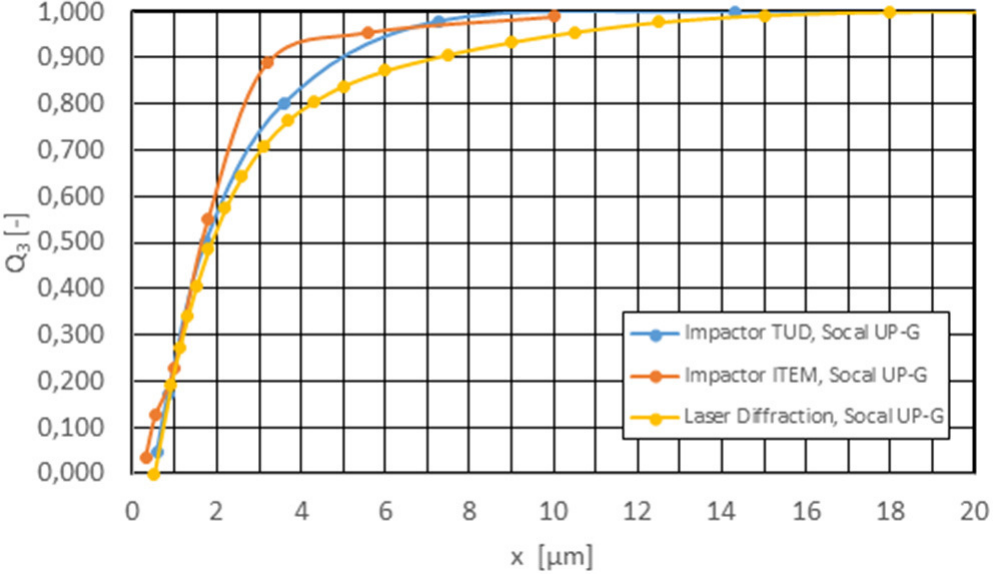
Comparison cascade impactor—laser diffraction for calcium carbonate after 3 h 30 min test duration; c_m_ = 500 mg/m3, ρ_eff_ = 1,000 kg/m^3^.

Figure 3 shows good agreement between the impactor results and the laser diffraction analysis using the example of the calcium carbonate system, which has good dispersibility and no tendency to agglomerate. The effective density of the particles can be estimated from the tap density of 447 kg/m3 at an assumed bulk porosity, the substance not tending to agglomerate, of 0.5 with about 1,000 kg/m^3^. This value was confirmed by our own impactor measurements.

**Figure 4:**
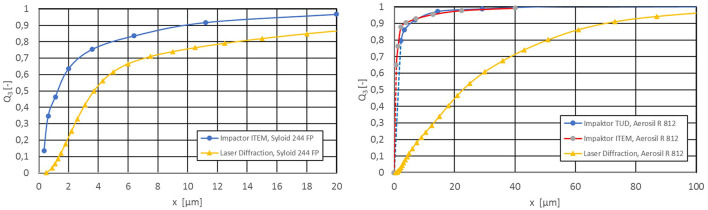
PSD - Comparison of cascade impactor and laser diffraction after an operating time of 3 h 50 min: left: silica gel, 500 mg/m3, ρ_eff_ = 242 kg/m3; right: surface-treated pyrogenic silica, c_m_ = 500 mg/m3, ρ_eff_ = 64 kg/m3 (tamped density).

In the case of aerosols which tend to form fragile agglomerates, these will be expected to be at least partially destroyed in the impactor due to shear stresses. Low-density powders, on the other hand, can have adhesion problems on the impactor plates due to the low particle density because a large particle volume must be deposited for a weighable mass. Measurements with silica gel and surface-treated pyrogenic silica therefore show finer measurement results, as expected (Figure 4).

In the case of silica gel, the cascade impactor provides values in the range of the known aggregate size distribution (Figure 4, left). The laser diffraction measurement also detects some coarser agglomerate structures due to the shear-free measurement. This effect occurs very clearly with pyrogenic powders, here the surface-treated pyrogenic silica, and provides unrealistic values as fragile agglomerate structures are largely destroyed in the aerosol stream insight the cascade impactor (Figure 4, right). In this case, the measurement results of the cascade impactors are complete unrealistic. For this reason, all measurements for aerosol characterization were subsequently carried out using laser diffraction spectroscopy.

### Particle Losses and One Worst-Case Example

**Figure 5:**
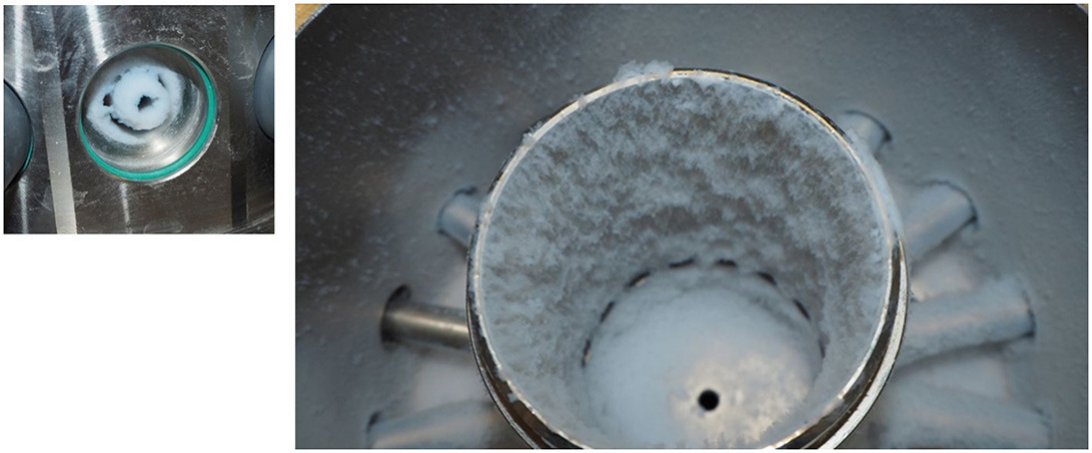
Untreated pyrogenic silica, particle deposits in the inhalation chamber after an operating time of 120 min with c_m_ = 4,700 mg/m^3^, left: exposure port, right: inner cylinder and chamber.

The particle size distributions shown in Figure 5 are laser diffraction measurements represented as a volume distribution of the geometric diameter. All measurements were made on the set-up as shown in Figure 2.

The worst-case example of a pyrogenic powder is used to illustrate the problems that can occur when high concentrations of powder aerosols are applied. Figure 5 shows the deposits in the inhalation system after exposure to an untreated pyrogenic silica aerosol - mass concentration at the aerosol generator 4,700 mg/m^3^ - over a period of 120 min. The deposits are clearly identifiable as agglomerates both in the inhalation chamber and at the exposure port.

**Figure 6:**
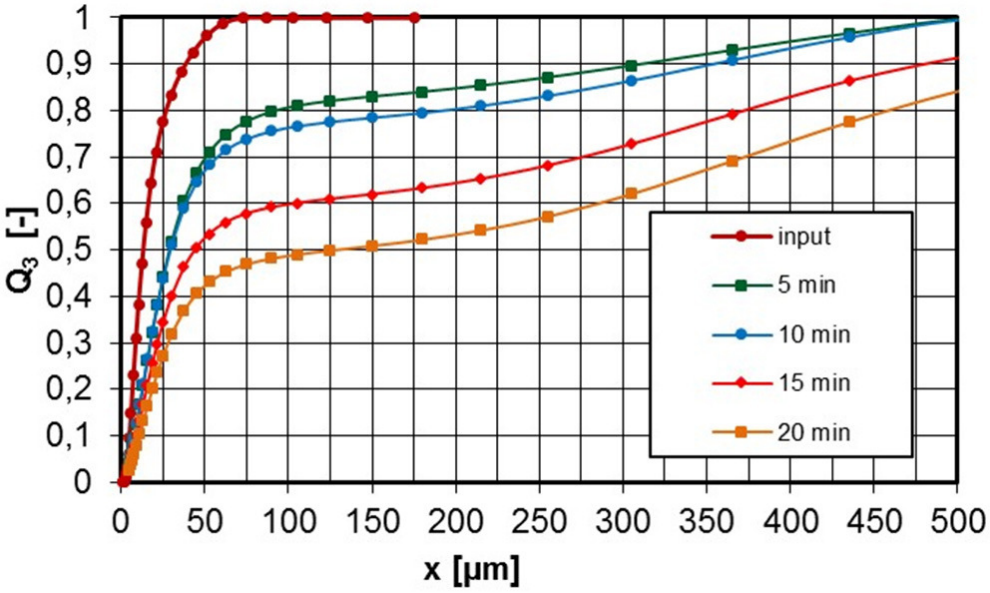
PSD at the inlet and exhaust tube of the inhalation chamber depending on the operating time, untreated pyrogenic silica, c_m_ = 4,700 mg/m^3^.

By using a laser diffraction meter at the inhalation chamber outlet, it was possible to detect even very large agglomerates. As shown in Figure 6, the aerosol already changes so much in the first minute of system operation that after 20 min the volume fraction of particles larger than 100 μm is already 50%. Under these conditions, an inhalation test would not be at all successful.

**Figure 7:**
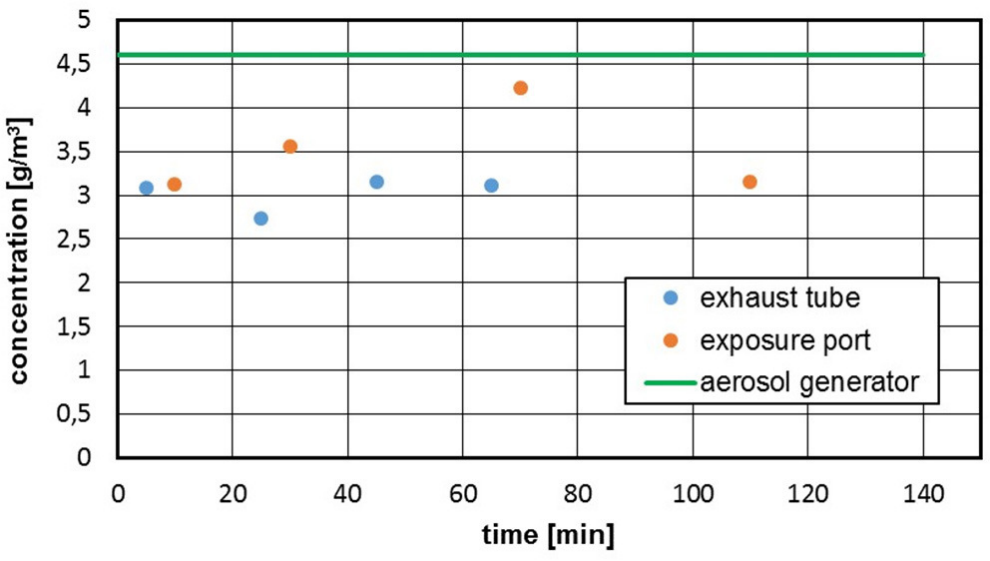
Untreated pyrogenic silica, c_m_ = 4,700 mg/m^3^, gravimetric mass concentration after different run times; green line: at the aerosol generator.

The particle deposits in the entire apparatus reveal the fact that the mass concentration both at the inhalation port and at the inhalation chamber outlet is significantly below the supplied concentration (Figure 7).

The mass losses in the inhalation test system depend on the powder material, the concentration and the test conditions. Mass losses in the range of 20–50% were determined for the investigated materials. These losses can be estimated in a preliminary test and readjusted at the aerosol generator. However, especially in the case of powders with a low bulk density and necessarily high aerosol concentration, there is a risk that the system can become heavily clogged within a short time and that the deposits can penetrate as far as the inhalation port.

### Comparison of Aerosol Condition at Different Concentrations

Experimental results are presented below for all investigated materials, documenting the geometric particle size distributions at the outlet of the inhalation system as a function of the operating time for two mass concentrations in each case. The concentrations given correspond to the values at the aerosol generator.

**Figure 8:**
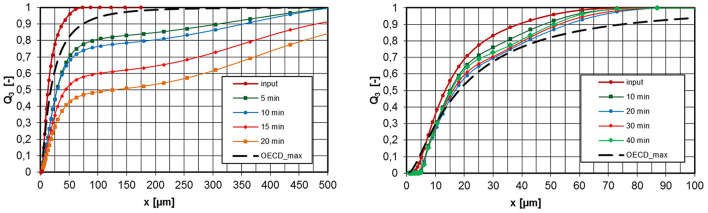
PSD at the inlet and exhaust tube of the inhalation chamber for different operating times, untreated pyrogenic silica, ρ_eff_ = 46,7 kg/m^3^ (tamped density), left: c_m_ = 4,700 mg/m^3^, right: 500 mg/m^3^.

Figure 8 shows measured values for aerosols from untreated pyrogenic silica at concentrations of 4,700 and 500 mg/m^3^. As already described, the measurements were taken at the aerosol generator inlet and at the exhaust tube after the aerosol had passed through the entire inhalation system for different operating times of the apparatus. In addition, the maximum permissible PSD, OECD_max_, according to OECD Guideline 436 is entered according to Figure 1. The conversion into an aerodynamic diameter is again carried out with Equation 1 based on the effective particle density ρ_eff_. If the measured PSD lies to the left of the OECD_max_ curve, the PSD meets the requirements for an inhalation experiment.

The left diagram in Figure 8 shows that for an aerosol concentration at the apparatus inlet of 4,700 mg/m3 there is already a volume-related agglomerate fraction of 20% >100 μm after 5-min operating time. According to the OECD_max_, the permissible fraction >100 μm would be about 5%. After 20-min operating time, this proportion increases further to >50% and more than 10% of the particle volume is >500 μm. Inhalation tests under these conditions are invalid because different problems can occur, e.g., missing inhalable fraction or blockage of the upper respiratory tract (nasal cavities). If the aerosol concentration is reduced to 500 mg/m^3^, the agglomerate formation is significantly reduced and the OECD_max_ specification was met over the investigated period of 40 min.

**Figure 9:**
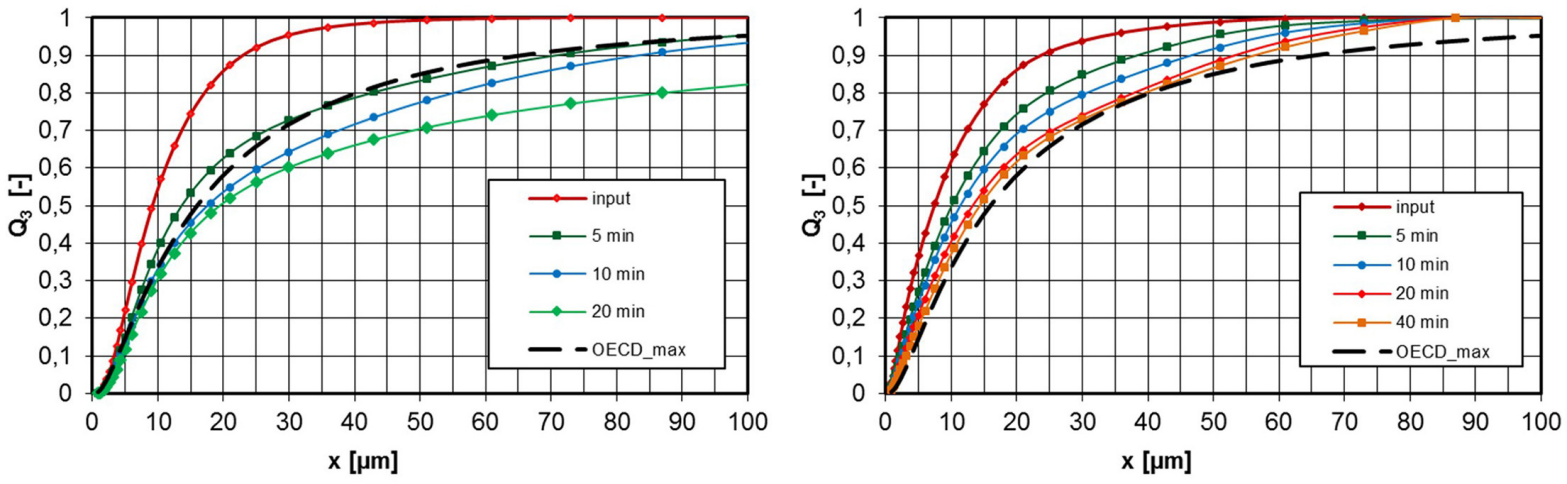
PSD at the inlet and exhaust tube of the inhalation chamber for different operating times, HMDS surface-treated pyrogenic silica, ρ_eff_ = 64 kg/m^3^ (tamped density), left: c_m_ = 5,100 mg/m^3^, right: 500 mg/m^3^.

A seconds pyrogenic powder, HMDS surface-treated pyrogenic silica was investigated in the same way. At a concentration of 5,100 mg/m3, the PSD is already outside the OECD_max_ specification after 5 min (Figure 9, left). As expected, the lower concentration of 500 mg/m^3^ showed a smaller change in the PSD due to agglomeration. Therefore, the specification was met over a period of 40 min.

**Figure 10:**
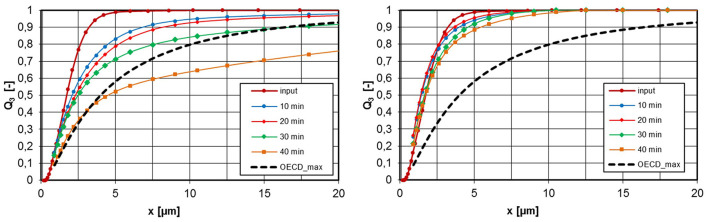
PSD at the inlet and exhaust tube of the inhalation chamber for different operating times, calcium carbonate, ρ_eff_ = 1,000 kg/m^3^, left: c_m_ = 6,300 mg/m^3^, right: 500 mg/m^3^.

Due to the higher primary particle density and the less fractal and coarser particle structure, as shown in Figure 10 for calcium carbonate, wet-chemically produced powders exhibit a lower tendency to agglomerate in the aerosol phase than pyrogenic produced powders. Even at a concentration of 6,300 mg/m^3^, the calcium carbonate aerosol was within specification for 30 min. At 500 mg/m^3^ a very stable aerosol atmosphere could be detected.

**Figure 11:**
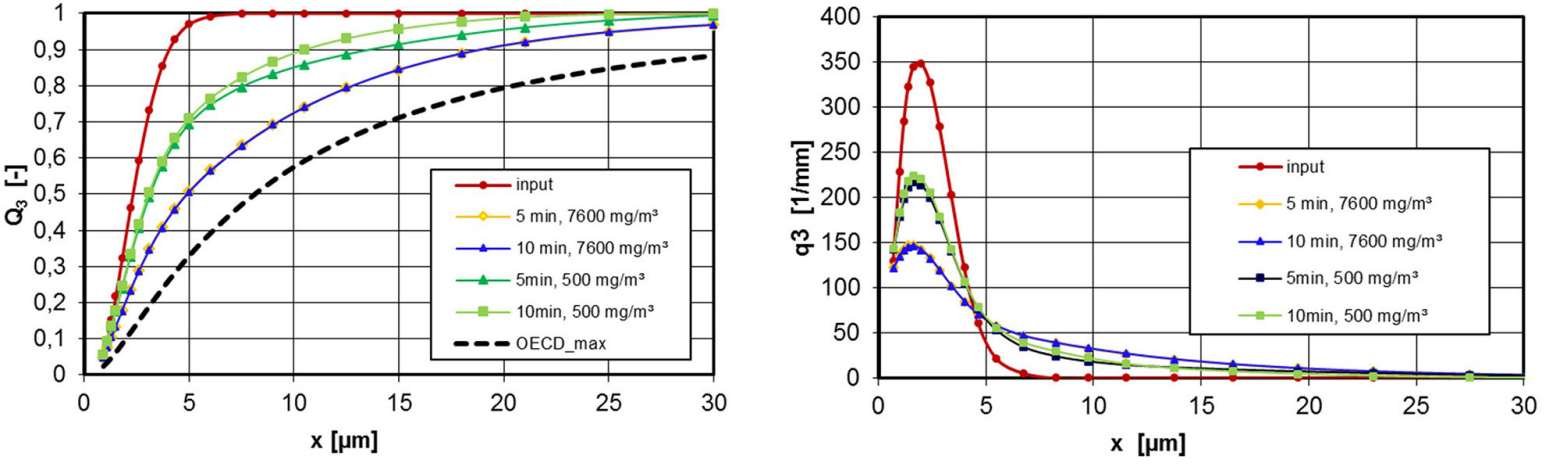
PSD at the inlet and exhaust tube of the inhalation chamber for different operating times, silica gel, ρ_eff_ = 242 kg/m^3^, left: c_m_ = 7,600 mg/m^3^ and 500 mg/m^3^, right: volume density distributions.

Figure 11 shows measured values of the silica gel powder, which is also produced by wet-chemical means (sol-gel process). The graph on the left shows the PSD for two aerosol concentrations in comparison with the OECD_max_ specification. The tendency to agglomerate depends on the concentration but is still acceptable for the high concentration in the period investigated. In the right graph of Figure 11, the measurement results are plotted as a volume density distribution. It can be clearly seen that part of the particle population (input) changes into an agglomerate population. The proportion of the aggregate population decreases with increasing agglomeration.

## Summary

In summary, it can be stated that the agglomeration of powder aerosols can significantly influence the results of acute inhalation tests. Strong agglomeration can lead to the absence of inhalable particles due to excessive particle sizes which could cause blockage of the inhalation ports. In either case, the test results would be unsatisfactory. However, even if the PSD of the aerosol is within specification according to OECD Guideline 436, agglomeration in the air stream can have a significant effect on the deposition site of the particles in the respiratory tract of the test animals, thus changing the effect of the aerosol and influencing the test result. Shang et al. (4) stated that particles of 3 μm geometric size are deposited to 100% in a rate nose model in the vestibule (the upper nose region) furthermore the velocity of the air is beside nose tip rather low and in the laminar region not inducing significant shear forces to the particles.

For the reasons mentioned above, it seems necessary to monitor the particle size of the aerosols from low density powders over the entire acute inhalation test period to reliably perform acute inhalation studies. For low-density powders (bulk density ≤ 100 kg/m^3^) used in this study, the use of cascade impactors has proven unsuccessful because the agglomerate structures are destroyed during the measurement. As an alternative and non-invasive measurement method, laser diffraction spectroscopy was very successful in the present investigations. With laser diffraction spectroscopy, concentrations from about 300 mg/m^3^ could be investigated. A significant advantage of this measurement method is that it can record the agglomeration tendency of a powder aerosol in real time and without shear.

From a metrological point of view, there is a need to develop an effective process sensor system that can detect and classify even the largest particle agglomerates as non-invasively as possible.

The particle measurement technology used should not destroy the fragile agglomerates that may form and must be capable to detect and ensure that the full size-range (measurement range) of low density powder aerosols is measured.

The maximum concentration that can be applied over 4 h depends to a large extent on the type of powder and should in any case be determined by preliminary tests. The operating conditions of the system, the concentration and the total test duration must all be considered. For the powder systems investigated in this study, it has been shown that concentrations up to 500 mg/m^3^ was technically feasible.

## Data Availability Statement

The raw data supporting the conclusions of this article will be made available by the authors, without undue reservation.

## Author Contributions

All authors listed have made a substantial, direct, and intellectual contribution to the work and approved it for publication.

## Conflict of Interest

The authors declare that the research was conducted in the absence of any commercial or financial relationships that could be construed as a potential conflict of interest.

## Publisher's Note

All claims expressed in this article are solely those of the authors and do not necessarily represent those of their affiliated organizations, or those of the publisher, the editors and the reviewers. Any product that may be evaluated in this article, or claim that may be made by its manufacturer, is not guaranteed or endorsed by the publisher.

## Figures and Tables

**Table:** 

**Symbol**	**Description**	**Unit**
c_m_	mass concentration	[mg/m^3^]
MMAD	mass median aerodynamic diameter	[μm]
q_3_	volume density distribution	[1/mm]
Q_3_	cumulative volume distribution	[-]
x	geometric particle diameter	[μm]
x_5_x_0_	median diameter	[μm]
x_ae_	aerodynamic particle diameter	[μm]
x_p;3_	particle size of the percentile p of Q_3_Q	[μm]
ε_agg_	agglomerate volume porosity	[-]
ε_p_	particle volume porosity	[-]
ρ_1000_	density = 1000	[kg/m^3^]
ρ_eff_	effective particle density	[kg/m^3^]
ρ_s_	true density of solid	[kg/m^3^]
σ_g_	geometric standard deviation	[-]
σ_ln_	logarithmic standard deviation σ_ln =_ ln(σ_g_)	[-]

## Abbreviations

CLP, Classification: Labeling and Packaging
HMDS: Hexamethyldisilazan
OECD: Organization for Economic Co-operation and Development
PSD: Particle size distribution.
